# Rapid Remote Online Evaluation in Endoscopic Diagnostics: An Analysis of Biopsy-Proven Respiratory Cytopathology

**DOI:** 10.3390/diagnostics13213329

**Published:** 2023-10-28

**Authors:** Hatice Elmas, Binnur Önal, Stefan Steurer, Birgit Hantzsch-Kuhn, Martin Claussen, Elnur Mehdi, Ümit Ince, Klaus F. Rabe, Guido Sauter, Lutz Welker

**Affiliations:** 1 Section Cytopathology, Institute of Pathology, University Medical Center Hamburg-Eppendorf UKE, D-20246 Hamburg, Germany; s.steurer@uke.de (S.S.); g.sauter@uke.de (G.S.); l.welker@gmx.net (L.W.); 2 Acıbadem Healthcare Group, Pathology Department, Acıbadem University, 34752 Istanbul, Turkey; binnurtr@gmail.com (B.Ö.); umit.ince@acibadem.com (Ü.I.); 3 LungenClinic Großhansdorf, D-22927 Großhansdorf, Germany; b.hantzsch-kuhn@lungenclinic.de (B.H.-K.); m.claussen@lungenclinic.de (M.C.); k.f.rabe@lungenclinic.de (K.F.R.); 4 Airway Research North (ARCN), Deutsches Zentrum für Lungenforschung (DZL), D-35037 Marburg, Germany; 5 Department of Nuclear Medicine, National Center of Oncology, 1012 Baku, Azerbaijan; elnur.mehdi@gmail.com

**Keywords:** telecytology, ROSE, telepathology, EBUS, lung cytology, endoscopic diagnostics, whole-slide imaging, diagnostic accuracy, cytopathology

## Abstract

Background: This prospective study assesses the use of rapid remote online cytological evaluation for diagnosing endoscopical achieved biopsies. It focuses on its effectiveness in identifying benign and malignant conditions using digital image processing. Methods: The study was conducted between April 2021 and September 2022 and involved analyses of 314 Rapid Remote Online Cytological Evaluations in total (154 imprint cytologies, 143 fine needle aspirations and 17 brush cytologies) performed on 239 patients at the LungenClinic Grosshansdorf. During on-site evaluation via telecytology, the time requirement was recorded and the findings were compared with the cyto-/histological and final diagnoses. Results: By means of rapid remote online evaluation, findings of 86 cytological benign, 190 malignant and 38 unclear diagnoses were recorded (Ø assessment time, 100 s; range, 11–370 s). In 27 of the 37 specimens with unclear diagnoses, the final findings were malignant tumours and only 6 were benign changes. The diagnosis of another 4 of these 37 findings remained unclear. Excluding these 37 specimens, rapid remote online evaluation achieved a sensitivity of 90.5% with a specificity of 98.5% and a correct classification rate of 92.4% with regard to the final diagnosis of all cases. As expected, an increase in the sensitivity rate for the cytological detection of malignant tumours (76.1% vs. 92.5%) was found especially in fine-needle aspirations. Conclusions: Rapid remote online analysis allows the fast quantitative and qualitative evaluation of clinically obtained cytological specimens. With a correct classification rate of more than 93%, sampling deficiencies can be corrected promptly and diagnostic and therapeutic approaches can be derived.

## 1. Introduction

Endosonography and targeted therapies have decisively changed bioptic and morphologic diagnostics. A range of detailed information is available on tumour type, histogenesis and spread patterns. At the same time, there is increasing interest in both cost- and time-saving, less invasive biopsy techniques, as well as in a reduced number of repeat biopsies and exploratory surgical procedures. In this context, rapid staining allows both an assessment of the quality and suitability of biopsy-derived samples for the further diagnostic and/or therapeutic measures required [1,2,3,4]. Different biopsy and processing methods are suitable for cytological rapid staining procedures [1,5,6,7,8,9,10,11]. Typically, rapid cytology examinations are performed as rapid on-site evaluations (ROSEs) [8,12,13,14]. As a rule, ROSEs are cost-intensive and rely on the on-site availability of personnel and equipment [1,8,15,16,17,18,19,20].

Cytopathology has undergone a rapid digital transformation in recent years, but it also faces various challenges related to image quality, scanning speed/method and storage issues. To solve these problems, different microscopes have been developed, and categorised according to the purpose by taking advantage of different technological infrastructures [15,16,17].

In contrast, online evaluation procedures not only enable the bridging of spatial distances between endoscopy units in hospitals and pathology institutes but also allow the morphologist to advise on/guide bioptic diagnostics. Remotely controlled procedures and transmission technologies enable rapid cytological analysis in real time and at the required scale [8,21,22]. The technical development of rapid remote online evaluation is a prerequisite for biopsies accompanying cytological assessments of tissue samples obtained on-site and stained rapidly [1,5,19,23].

Further, in biopsy diagnostics, it is possible to bridge spatial distances between endoscopy units in hospitals and pathology institutes using remote procedures and transmission technologies, thus enabling rapid cytological analyses to be conducted to the desired extent [8,21,22].

The present study aims to record the suitability of motorised real-time cytology for endoscopic biopsy diagnostics. For this purpose, the time required and the sensitivity and specificity of the rapid remote online evaluation were recorded during the examination of 239 patients at the LungenClinic Großhansdorf and finally compared with the present final diagnosis.

## 2. Material and Method

**Figure 1:**
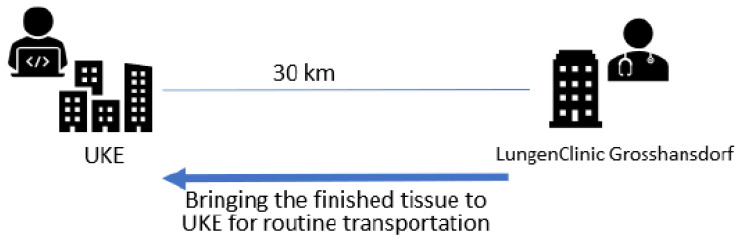
Technical implementation—locations: LungenClinic Großhansdorf (**right**) and Institute of Pathology University Medical Center Hamburg Eppendorf (UKE) Hamburg (**left**). This map illustrates the distance of about 30 km between the two sites.

The tissue samples were taken in the endoscopy unit of the LungenClinic Großhansdorf and cytological examinations were performed at the distance of 30 km from the Institute of Pathology of the University Medical Center Hamburg Eppendorf (UKE Hamburg, Figure 1). All samples obtained, including the rapidly stained preparations were transported over an average transport time of one hour and ten minutes to the UKE Hamburg for final processing.

Between 4/2021 and 9/2022, 239 patients (131 m and 108 f; mean age, 67.8 (range: 22–89 years) underwent 314 rapid remote online evaluations in total (17 brush cytologies, 143 fine-needle aspirations and 154 imprint cytologies, Table 1, Table 2, Table 3 and Table 4). Patients were included if their clinical and imaging results were promptly obtained; cytological findings of uncertain biological potential, an insufficient number of diagnostical cells, and discrepancies between a clinical diagnosis of suspicion and cytological findings (the wrong sampling site) were named lesions of undefined biological behaviour (Table 5, Table 6, Table 7 and Table 8). Material collection and Giemsa rapid staining were performed in the endoscopy department by clinically active endoscopists/assistant staff.

**Figure 2:**
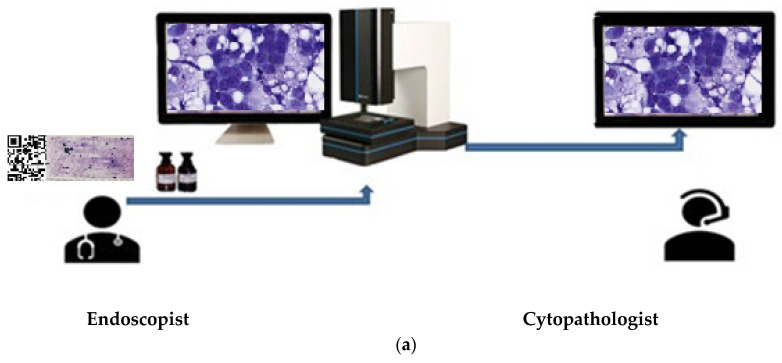

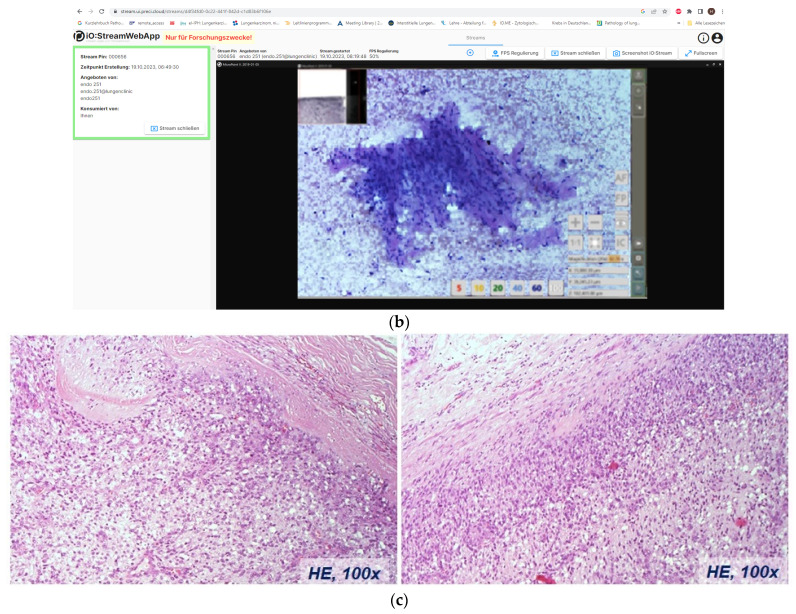
(**a**) Rapid remote online evaluation technical procedure. (**b**) EBUS-FNA of an intimal sarcoma of a right pulmonary vein associated with the involvement of the right atrium; 22-year-old, non-smoker (rapid remote online evaluation analysis; time requirement: 2 min. for malignant cyto-diagnosis in favour of a mesenchymal tumour, in light of imaging and clinical findings). (**c**) Histological diagnosis of the intimal sarcoma case of the right pulmonary vein associated with the involvement of the right atrium; the same case as in (**b**).

**Figure 3:**
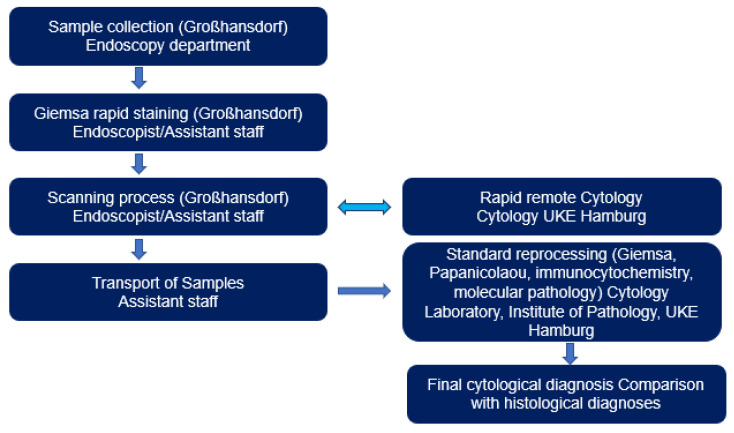
Rapid remote online evaluation workflow for the evaluation of endoscopically obtained specimens at the LungenClinic Großhansdorf and their rapid online evaluation up to physical transport and final cytological evaluation at the Institute of Pathology UKE Hamburg.

For the evaluation of the images, one monitor each was available at each site. The specimens were stained during the ongoing endoscopic examination, using iO:M8 Digital Live Microscope from PreciPoint (Freising, Germany; ISO 13485:2016 certified) and viewed for first time once a telephone/internet connection was established. Discussion of findings and evaluation were performed between clinically and morphologically active colleagues via telephone (Figure 2 and Figure 3). At the same time, representative images of the respective cases were stored by the cytological investigator. When delivering the findings to the endoscopists during the rapid procedure, it is essential to carry out the following: (1) explicitly indicate whether a lesion or tumour is present or absent in the sample or resected specimen, (2) provide information on whether the lesion is determined to be benign or malignant, or if its malignancy status remains undetermined, and (3) convey whether there is a requirement for further sampling or if the sample should be sent to the microbiology/flow cytometry/immuno-molecular diagnosis section. The time required in each case for the morphological assessment of rapid remote online cytology was measured. Simultaneously, for each case, two typical images describing the case were taken and archived.

The mean duration of cytological examination following methanol fixation, Giemsa staining and the rinsing procedure was 100 s (range: 11–370 s).

After transport to the cytology laboratory of the Institute of Pathology of the UKE Hamburg, all samples were subjected to standard Giemsa and/or Papanicolaou staining. Where necessary, Papanicolaou-stained preparations were overstained for immunocytochemistry, and Giemsa- and Papanicolaou-stained preparations were used for molecular analyses (reprocessing). The cytological rapid findings were than compared with all available results of the morphological findings of the individual patients obtained at the same time or subsequently. A multidisciplinary approach was established for the definitive diagnosis, incorporating clinical radiology, histopathology or clinical cytopathology findings.

iO:M8 Digital Live Microscope enables the real-time transmission of high-resolution microscopy images and secure remote control of the connected microscope by authorised on-site personnel simultaneously. It draws on the possibilities of Web Real-Time Communications (WebRTC) for real-time communication between clients on the web.

The system supports a wide range of devices, including workstations and laptops, provided they are equipped with a Chromium-based, WebRTC-supporting browser. Transmission resolutions can vary from high definition (1920 × 1200) to 4K (3840 × 3840) to adapt to different bandwidth requirements. An adjustable frame rate between 15 and 60 FPS ensures performance with connections between 15 and 80 Mbps.

A web-based system operating within a secure institutional network, iO:M8 Digital Live Microscope, combines user-friendly accessibility with high speed and data security.

## 3. Results

Within a mean assessment time of 100 s, 314 rapid remote online cytology evaluations were performed in total. The success of a rapid online cytology remote evaluation is influenced by the level of training and experience of the clinical-bioptical endoscopist as well as the clinical–morphological knowledge of the cytologist. The morphological interpretation of the transmitted image content requires, in addition to the specific endoscopic findings, comprehensive knowledge of previous clinical anamnestic data and other imaging findings. The present study includes the analysis of 17 brush cytologies, 143 fine-needle aspiration cytologies and 154 imprint biopsies. Table 3 and Table 4 demonstrate the number of rapid remote online evaluations—findings versus the nature of the determination of the final diagnosis and the distribution of the final diagnoses of the 239 patients. The selection of the consecutively examined patients and the indication for rapid examination were carried independently from the morphologist exclusively by the working clinical endoscopists.

Based on rapid remote online cytology, 86 benign, 190 malignant, and 38 undefined diagnoses were recorded. Briefly, 27 of the 37 specimens were final malignant tumours and only 6 were benign changes. The diagnosis of another 4 of these 37 specimens remained unclear (Table 5). Of the 37 undefined samples, 11 were still unclear at the end according to the cytological criteria and 4 of these were unclear according to histological criteria. One major reason for the large number of unclear samples is the lack of representative cell and tissue fragments. The results of the rapid cytological assessment prompted the endoscopists in this situation to intensify biopsy diagnostics and to perform repeated biopsies per sampling site. Accordingly, the number of inconclusive biopsies was reduced to six in the end (Table 5).

Following the completion of diagnostics and excluding the results with undefined diagnosis, there were finally 71 specimens classified as benign changes and 237 as malignant tumours based on the final diagnosis of all findings (Table 5). In six specimens, the final diagnosis could not be determined. If the finally undefined observations were excluded, then a sensitivity of 80.2% was achieved with a specificity of 100% and a correct classification rate of 84.7%.

Cohen’s kappa was run to determine if there was agreement between the remote online evaluation tests and final diagnoses. There was moderate agreement between the two test results, with κ = 0.52 (95% CI: 0.49; 0.56) and *p* < 0.001. If we exclude the undefined 37 specimens (n = 277), there is very good agreement between the two test results, with κ = 0.82 (95% CI: 0.78; 0.86) and *p* < 0.001 (Table 5).

After the standard processing of cytological specimens (via Papanicolaou and Giemsa histochemical staining and immunocytochemistry), the number of undefined findings decreased to 13 cases. As expected, sensitivity increased especially with fine-needle aspirates (76.1% vs. 92.5%. Table 7 and Table 8). Cohen’s kappa was run to determine if there was agreement between the cytological analysis and final diagnosis. There was strong agreement between the two test results, with κ = 0.71 (95% CI: 0.67; 0.75) and *p* < 0.001. If we exclude the undefined results of the assessment, there is almost perfect agreement between the two test results, with κ = 0.87 (95% CI: 0.83; 0.90) and *p* < 0.001.

In 131 of 227 findings (57.7%), the tumour types observed via rapid remote online cytology matched the final diagnoses. The highest match rate was found for neuroendocrine-differentiated tumours (35 of 47 tumours, corresponding to 74.5%, Table 9).

## 4. Discussion

Our study highlights the difficulties of rapid remote online evaluation for ROSEs with years of experience and highlights the assessment timing (n = 314) [19,21]. Between January 1999 and December 2020, 252,467 biopsies in total were obtained at the LungenClinic Großhansdorf. Clinical colleagues endoscopically obtained 79.001 FNA, 28.731 brush cytologies and 55.294 touch preparations. The cytological expertise was based on this examination material and especially on 22.781 ROSE evaluations.

For comparison, our sample size was sufficient for an accurate diagnosis. We demonstrated the effectiveness of our rapid remote online evaluation application by comparing the images seen through the process and the skill assessment conducted by the final cytopathologist when all materials were ready for evaluation. In our series, the overall diagnostic accuracy was 96.7%. This matches our previous physical on-site evaluation rates and previous reports that showed an agreement rate of 80% to 95% for Rapid Remote Online Evaluation and 66.7% to 97% for traditional on-site methods [5,8].

Groundbreaking advances in molecular biology have led to the development and establishment of targeted therapy and thus to a dramatic improvement in the oncological treatment of lung cancer patients. Nevertheless, the prerequisite for the appropriate treatment of malignant tumours mostly remains confirmation through morphology.

Rapid cytological evaluation procedures such as ROSE or rapid remote online evaluation initially allow an assessment of the quantitative and qualitative suitability of obtained cellular samples for further required standard and immunochemical analyses, as well as for necessary molecular pathological investigations [24,25,26]. In the end, rapid cytological analyses are expected to reduce the number of repeated endoscopic examinations, along with the associated costs and biopsy-related risks. The final result is expected to be an optimisation of the time window between clinical manifestation and targeted tumour therapy. Figure 2b demonstrates an example of a 22-year-old young man with an advanced tumour of the right pulmonary vein, extending into the right atrium. The type of tumour presents significantly influences the extent of resection. The use of rapid remote online evaluation considerably shortened the time required for diagnosis and contributed to the avoidance of an otherwise necessary thoracic surgical intervention to confirm the type of tumour present. Knowing the rapid remote online evaluation findings, a pneumonectomy and partial atrial resection were performed without any delay. Histologically, the tumours were intimal sarcomas of the right pulmonary vein. Unlike all previously published rapid assessment methods, the concept presented here relies on close cooperation in both sample collection, processing, and assessment. The rapid remote online evaluation is not an anonymous magic black box where one sends something and receives a result; instead, it demands an interactive cognitive process. Clinical–bioptic and morphological expertise is shared between the endoscopist and morphologist, fostering a mutual learning process on both sides.

Endoscopic biopsy diagnostics is a complex, invasive and cost-intensive procedure. The learning process for cytotechnologists/cytologists is long and difficult. The number of cytologically skilled morphologists is very small. The learning curve for cytotechnologists/cytologists is also steep and time-consuming.

Since the pool of skilled cytopathologists/cytologists and cytotechnologists is limited, the primary goal of this concept is to make cytological expertise accessible, efficient and cost-effective, especially for smaller hospitals with limited personnel and financial resources [23,27]. With regard to biopsy-accompanying rapid examinations, ROSE has so far established itself as a standard procedure. Two questions are of central interest for the use of ROSE [5,11,12,28]:

1. Is the biopsy material obtained qualitatively adequate for the fine tissue changes present at the target site?
2. Is the material quantitatively sufficient for all necessary further analyses including molecular pathological procedures?

Material is collected in the presence of cytotechnologists/cytologists. The latter perform rapid staining and an initial analysis and in the case of telecytology, send selected images to a pathology institute for evaluation. The associated logistical and personnel costs, limited resources and lack of remuneration for such solutions hinder widespread coverage [5,29,30].

Deviating from this, the rapid staining in rapid remote online evaluation is performed by assistants/endoscopists. The simultaneous optical evaluation of specimens by morphologically experienced examiners and endoscopically active clinical colleagues is a special feature of the solution presented here. Clinical colleagues are finding it increasingly easier to enhance their own biopsy expertise independently and to influence both the quantity and invasiveness of subsequent biopsy procedures [12]. The abundance of detailed clinical information available simplifies the morphological assessment for cytotechnologists/cytologists. The lack of a physical presence and delays due to waiting times in the endoscopy procedure limit the interruption of the cytotechnologist/cytologist’s routine activity only for the absolutely necessary very short assessment time of about 100 s. In principle, various advantages and disadvantages can be expected with rapid remote online evaluation (Table 10 and Table 11). Time measurement was conducted automatically by the system while performing a Rapid remote online **evaluation** of the patient whose clinical–radiological findings were **already** known.

Methodologically, rapid remote online evaluation is comparable to physical rapid cytology on-site evaluation—ROSE—in terms of potential challenges and limitations. Essentially, as with all small tissue samples, a quantitative mismatch between tumour and sample size can be anticipated. This mismatch is likely to be a major cause of divergent tumour type diagnoses (Table 9).

Rapid examinations are inherently operated under time constraints. Diagnoses rely on the assessment of specific small areas. Immunocytochemical evaluation criteria are not accessible during this process. Typically, temporary nucleolar overstaining occurs initially during rapid staining, in contrast to standard staining. Additionally, the cytoplasm of tumour cells is often suboptimally stained. An assessment of thick and/or poorly stained areas remains difficult in the time available. If necessary, brightness and image sharpness must be readjusted depending on the material thickness of the preparations.

The diagnostic yield of rapid remote online evaluation analyses is very credible with a correct classification rate of more than 93% (Table 5 and Table 6). In addition, even tumour-suspicious and/or negative rapid remote online evaluation findings may adequately reflect the structure and/or nature of the tissue at the biopsy site. If a cytological finding deviates from the clinically expected result, either repeated biopsies are promptly ordered or the suspected clinical diagnosis is changed during endoscopy. However, if more than one specimen is obtained at a biopsy site, rapid remote online evaluation and standard assessment results may differ. In particular, this is a characteristic constellation of endo-sonography-guided per bronchial EBUS-FNA. This showed an increase in sensitivity for rapid remote online evaluation and standard analyses from 76.1% to 92.5%. However, sensitivity is first of all a characteristic of the suitability of a biopsy procedure to obtain adequate tissue and not a suitable evaluation criterion for morphological analyses obtained post-factum. The level of sensitivity thus documents the degree of (bioptic manual) skill of an endoscopist in selecting and using a suitable biopsy procedure. If no tumour cells are present in a tumour punctate, the cytopathologist may not be able to detect a malignant tumour. It is unrealistic to expect changes in image content from imaging techniques. An increase in the sensitivity of samples cannot be achieved via either ROSE or rapid remote online evaluation but only via repeated biopsies [8]. Conversely, in the hands of experienced endoscopists, ROSE does not increase diagnostic yield but only decreases the number of successful needle passes during EBUS-TBNA according to Sehgal IS et al., 2018 [7].

In contrast, specificity, i.e., the ability of cytological methods to distinguish benign findings from malignant tumours, is completely independent of the influence of the endoscopist and exclusively characterises the performance of the cytopathologist. If tumour cells are contained in a material and the cytopathologists/cytologists is unable to reliably assign them to a malignant tumour, the number of cases correctly identified decreases.

Telecytology is the remote interpretation of cytological material using digital images. Most telecytology studies in the literature focus on the review of static digital photomicrographs or video microscopy, which allow the remote viewer to assess only a tiny fraction of the entire case material. It is also possible to scan entire slides. Whilst the solutions used to date have limited the remote viewer’s ability to select images and either see only a small fraction of the material on the images, the time taken to scan the whole slide and the assessment required is too long for biopsy-accompanying solutions. Endoscopic biopsy procedures are significantly more demanding compared to established surgical procedures for obtaining specimens for frozen section studies. Comparable to frozen section diagnostics, however, the rapid remote cytological diagnostics presented here are not primarily aimed at a conclusive detailed morphological diagnosis, but rather at the acquisition of selected bioptically and diagnostically relevant contents. Error-prone specimen collection, slide preparation, fixation and staining are performed under the guidance of clinical partners. Remotely controlled slides allow (dynamic) simulated microscopy in real time. The joint evaluation of slides on the screen by endoscopists and cytopathologists in real time allows the simultaneous detection of technical sampling and processing errors as well as diagnostically relevant cell changes. As a result, sample collection can be technically adjusted, promptly terminated or escalated if necessary. Accordingly, the solution presented here is aimed at both optimising collection quality and improving diagnostic yield.

## 5. Conclusions

Based on these rapid remote online evaluation findings, relevant diagnostic and therapeutic decisions can be derived in a timely manner. A quality-assured rapid remote online evaluation process enables an assessment of the quantitative and qualitative suitability of obtained cellular samples for further required standard and immunocytochemical analyses, as well as necessary molecular pathological examinations [8,18,21]. An interdisciplinary understanding of the clinical problem and the morphological facts reduces friction among disciplines and is an essential prerequisite for customised diagnostics.

## Figures and Tables

**Table 1:** Gender and age distribution of the study population.

Distribution of Patients (Gender, Mean Age Range)	*n* (%)	Mean Age (Range)
Female	108 (45.2%)	68.7 (33–88)
Male	131 (54.8%)	66.9 (22–89)
Total	239 (100%)	67.8 (22–89)

**Table 2:** Distribution of the examined specimens (*n* = 314).

Sampling Materials	*n* (%)
Fine-needle aspirations (EBUS/EUS FNA)	143 (45.5%)
Imprint (Touch) preparations	154 (49.0%)
Brush cytologies	17 (5.4%)
Total	314

**Table 3:** Number of rapid remote online evaluations—findings versus nature of determination of final diagnosis.

Nature of Determination of the Final Diagnosis	*n*
Clinical–imaging–cytological *	53
Histological	261
Total	314

**Abbreviations:** * Clinical dx: medical history, clinical findings, and endoscopy; imaging: X-ray, CT, PET-CT, and MRT.

**Table 4:** Distribution of the final diagnoses of the patients (*n* = 239).

Histologic Type	*n* (%)
Primary lung cancer	
Adenocarcinoma	68 (28.5%)
Squamous cell carcinoma	54 (22.6%)
SCLC	34 (14.2%)
Carcinoid/neuroendocrine tumour of the lung	4 (1.7%)
Large cell neuroendocrine carcinoma	2 (0.8%)
Non-small cell carcinoma, NOS	17 (7.1%)
Other malignant tumours	-
Sarcomas	3 (1.3%)
Malignant non-Hodgkin lymphoma/CLL, mature B-Cell neoplasm (CLL)	3 (1.3%)
Mesothelioma	2 (0.8%)
Breast carcinoma	3 (1.3%)
Endometrial carcinoma	1 (0.4%)
Cervical carcinoma	1 (0.4%)
Urinary bladder carcinoma, urothelial carcinoma	1 (0.4%)
Benign diseases	43 (18.0%)
Undefined morphological differentiation/biological behaviour	3 (1.3%)
Total	239

**Abbreviations:** *n*—total number of patients; SCLC—small cell lung carcinoma; NOS—not otherwise specified; CLL—chronic lymphocytic leukemia. **Benign diseases:** Squamous metaplasia, inflammatory-reactive changes in the lung and bronchial mucosa, reactive lymphadenitis, severe purulent inflammation, abscesses, and epithelioid cell granulomatosis.

**Table 5:** Rapid remote online evaluation vs. final diagnosis.

Rapid Remote Online Evaluation Diagnosis	Final Diagnosis			Total
	Malignant	Benign	Undefined	
Malignant	190	0	0	190
Benign	20	65	1	86
Undefined	27	6	5	38
Total	237	71	6	314

**Undefined:** necrosis, dysplasia, unrecognisable direction of differentiation or undefined biological behaviour, insufficient number of diagnostic cells, and discrepancy between clinical diagnosis of suspicion and cytological findings (wrong sampling site).

**Table 6:** Rapid remote online evaluation vs. final diagnosis: sensitivity, specificity and diagnostic accuracy rate (**all cases: *n* = 314 and *n*_1_ = 276; 38 cases of undefined diagnosis excluded**).

	*n*	Sensitivity (%)	Specificity (%)	Diagnostic Accuracy (%)
Brush cytology	15	100	100	100
Fine needle aspiration cytology	120	76.1	100	89.9
Imprint cytology	142	93.0	96.4	93.7
Total	277	90.5	98.5	98.5

**Table 7:** Standard cytological analysis vs. final diagnosis (***n* = 314 specimens**).

Standard Cytology	Final Diagnosis			Total
	Malignant	Benign	Undefined	
Malignant	222	3	2	227
Benign	7	69	2	78
Undefined	6	1	2	9
Total	235	73	6	314

**Undefined:** necrosis, insufficient number of diagnostic cells andsmall connective tissue fragments in mesenchymal tumours of undefined biological behaviour.

**Table 8:** Standard cytological diagnosis vs. final diagnosis. Sensitivity, specificity and diagnostic accuracy rates (all specimens: *n* = 314 and ***n*_1_ = 303**; 11 findings of undefined standard cytological diagnosis excluded).

	*n*	Sensitivity (%)	Specificity (%)	Diagnostic Accuracy (%)
Brush cytology	17	100	100	100
Fine needle aspiration cytology	135	99.0	100	99.3
Imprint cytology	151	97.5	100	98.0
Total	303	98.2	100	98.7

**Table 9:** Rapid remote online evaluation pre-diagnosis vs. final diagnosis of selected lung cancer types (*n* = 314 **specimens**; *n*_1_ = 227 **primary lung cancer patients**).

	*n*_1_	Rapid Remote Online Evaluation (in %)
Adenocarcinomas	87	43 (49.4%)
Squamous cell carcinoma	67	38 (56.7%)
Neuroendocrine neoplasms *	47	35 (74.5%)
Non-small cell carcinoma, NOS ^#^	26	15 (57.7%)
Total	227	131 (57.7%)

* **Neuroendocrine** neoplasms; carcinoid/NET, small-cell lung cancer and large-cell neuroendocrine carcinomas are included. ^#^ NOS: not otherwise specified.

**Table 10:** Advantages of rapid remote online Evaluation [19,23,31,32,33].

Advantages
Interactive mutual training of the endoscopist and orphologist on endoscopic, morphological findings and clinical issues
Decreased TAT and total around time for reporting and operative processes
More efficient use of human resources during rapid remote online evaluation procedure
Decreased costs of hospital resources
Increasing morphological competence of the endoscopist and clinical competence of the morphologist

**Table 11:** Disadvantages of rapid remote online evaluation [19,23,31,32,33].

Disadvantages
Examination of limited number of specimens obtained and restriction to selected areas (time pressure)
Cytoplasm staining is often suboptimal Temporary nucleolar over-staining occurs
Difficulty in assessing areas that are too thick and/or poorly stained
May require the readjustment of brightness and image sharpness
Potential technical problems such as internet access, password failure and remote medical technology system failure.

## Data Availability

The data that support the findings of this study are available from the corresponding author upon reasonable request.
